# A retrospective analysis of emergency department cultures in a tertiary care center

**DOI:** 10.1186/s12879-026-14015-w

**Published:** 2026-07-15

**Authors:** Nevin İnce, Güneş Şengüldür, Emel Akbaş, Erdinç Şengüldür, Dilek Akıncı, Bekir Tunca

**Affiliations:** 1 Department of Infectious Diseases and Clinical Microbiology, Düzce University Faculty of Medicine, Düzce, Türkiye; 2 Department of Medical Microbiology, Düzce University Faculty of Medicine, Düzce, Türkiye; 3 Department of Emergency Medicine, Düzce University Faculty of Medicine, Düzce, Türkiye

**Keywords:** Emergency department, Microbiological cultures, Contamination, Urine culture, Blood culture

## Abstract

**Aim:**

To describe the distribution and outcomes of cultures obtained in the emergency department (ED), examine contamination and microbiological yield across specimen types, and compare results between adult and pediatric settings.

**Methods:**

We conducted a retrospective study including all cultures obtained over a 12-month period in the adult and pediatric EDs of a tertiary hospital. Demographic characteristics, culture type, microbiological results, and patient disposition were extracted from electronic records. Culture results were categorized as no growth, contamination/commensal-flora, bacterial growth with or without extended-spectrum β-lactamase (ESBL) production, yeast growth, or sample rejection.

**Results:**

A total of 2,674 cultures were analyzed. Nearly half of the cultures showed no growth (48.8%), and 24.3% were classified as contamination/commensal-flora based on laboratory report wording, representing low-interpretability flora reports rather than a formal blood culture contamination rate. Bacterial growth without ESBL production occurred in 16.6%, while ESBL-producing organisms were detected in 2.3%; yeast growth occurred in 1.0%, and 7.0% of samples were rejected. Urine cultures constituted the largest proportion and demonstrated higher rates of both growth and contamination/commensal-flora compared with blood and sterile body fluid cultures. Contamination/commensal-flora was more frequent in pediatric ED cultures, whereas hospitalized—particularly intensive care—patients had higher microbiological positivity and a greater prevalence of ESBL-producing organisms.

**Conclusions:**

A substantial share of ED cultures yielded no growth or laboratory-wording–based contamination/commensal-flora reports, suggesting limited diagnostic interpretability in many cases. Variation by specimen type and patient disposition highlights the importance of selective culture ordering, improved collection practices, and integration of antimicrobial stewardship principles into ED workflows. Prospective multicenter studies incorporating clinical outcomes are needed to clarify the impact of ED culture practices on patient care.

## Introduction

Infectious diseases constitute a substantial proportion of emergency department (ED) presentations worldwide and are among the leading causes of morbidity, hospitalization, and healthcare expenditure [1, 2]. Patients frequently present to the ED with nonspecific systemic symptoms such as fever, malaise, and altered mental status, as well as localized complaints related to urinary, respiratory, intra-abdominal, or soft-tissue infections [3, 4]. Because diagnostic and therapeutic decisions are often made under time pressure and with limited initial information, early and accurate identification of the infectious source is critical for guiding appropriate management and preventing deterioration [3, 5, 6].

Microbiological cultures remain a cornerstone of diagnostic evaluation in suspected infection. Culture results help confirm or refute the presence of a pathogen, inform targeted antimicrobial therapy, and play a pivotal role in antimicrobial stewardship initiatives [6]. Inappropriate or delayed culture acquisition may result in unnecessary broad-spectrum antibiotic exposure, increased resistance, prolonged length of stay, and worse clinical outcomes. Conversely, timely and properly obtained cultures support rational antibiotic selection and improve patient safety [6, 7].

Despite their central importance, culture acquisition in the ED is prone to several procedural and organizational errors. Common pitfalls include inadequate aseptic technique, contamination during collection, inappropriate patient selection, and failure to obtain cultures before antibiotic administration [8]. Contaminated cultures may trigger false-positive results, leading to unnecessary antibiotic treatment, additional diagnostic tests, and prolonged hospitalization, whereas missed or poorly handled cultures may delay diagnosis and compromise care. Understanding the frequency and consequences of such issues in real-life ED practice is essential for quality improvement [8, 9].

Although previous ED studies have largely focused on blood culture contamination or selected specimen types, fewer real-world analyses have evaluated the overall operational burden of multiple culture types, including urine, blood, and sterile body fluid cultures, across both adult and pediatric ED settings and according to patient disposition. Therefore, this study aimed to describe the distribution and outcomes of microbiological cultures obtained in the ED, with particular attention to culture types, contamination/commensal-flora reporting, microbiological yield, sample rejection, and patient disposition, and to compare these patterns between adult and pediatric ED settings.

## Materials and methods

### Study design and setting

This retrospective observational study included all microbiological cultures obtained over a 12-month period (01.01.2025–01.01.2026) in the adult and pediatric EDs of a tertiary care hospital. Both EDs are staffed by board-certified emergency physicians and serve a mixed urban and regional population.

### Study population and data collection

All consecutive culture samples obtained from patients presenting to the adult or pediatric ED during the study period were screened. The inclusion criterion was the availability of at least one microbiological culture obtained during the ED visit between 01.01.2025 and 01.01.2026. Records with missing essential data required for analysis, such as age, sex, ED type, culture type, microbiological result, or disposition status, were excluded. No additional exclusion criteria were applied. Demographic characteristics (age and sex), ED type (adult vs. pediatric), culture type, microbiological results, and admission status at the end of the ED visit were retrieved from the electronic hospital records. All analyses were performed at the culture-sample level. Because cultures could recur in the same individual across different ED admissions, observations may not be fully independent.

### Culture classification and microbiological outcomes

Culture samples were grouped as urine cultures, blood cultures (aerobic and/or anaerobic), and sterile body fluid cultures. In routine ED practice, urine samples are generally obtained as midstream clean-catch specimens in cooperative adult patients, from urinary catheters in selected catheterized patients, and by urine collection bags in younger children when clean-catch sampling is not feasible. However, the exact urine collection method was not consistently recorded as a structured sample-level variable in the electronic records; therefore, urine cultures could not be stratified according to collection method. Patient disposition was categorized as discharge from the ED, ward admission, or intensive care unit (ICU) admission. Microbiology reports were extracted verbatim as issued by the laboratory. Prior to analysis, laboratory terminology was harmonized using a predefined dictionary solely to merge synonymous expressions (e.g., variants of “no growth”). We used a laboratory-wording–based proxy for contamination/commensal-flora reporting: any final report explicitly stating “mixed flora,” “commensal flora,” “normal skin flora,” “urogenital flora,” or similar wording indicating probable contamination was coded as contamination/commensal-flora. This category was intended to capture the operational burden of low-interpretability flora reports and should not be interpreted as confirmed blood-culture contamination as defined in quality-benchmarking studies. No investigator adjudication, reinterpretation, or post hoc re-classification was performed beyond the predefined terminology harmonization. Sample rejection was defined according to the final laboratory report. Specific reasons for rejection, such as inadequate specimen volume, inappropriate containers, leakage, labeling errors, delayed transport, or other preanalytical issues, were not consistently available as structured variables. Fungal findings captured in the available dataset were categorized as yeast growth. Filamentous fungi, such as Aspergillus spp. or Mucorales, were not recorded as a separate microbiological category; therefore, no separate filamentous fungal analysis could be performed.

### Statistical analysis

Continuous variables were summarized as median (25–75) and categorical variables as % (n). Normality was assessed using visual inspection (histograms and Q–Q plots) and the Shapiro–Wilk test. Comparisons between pediatric and adult ED cultures were performed using the Mann–Whitney U test for continuous variables and the Pearson chi-square test for categorical variables; when expected cell counts were < 5, exact tests were applied (Fisher’s exact test for 2 × 2 tables and exact/Monte Carlo methods for r×c tables). For comparisons involving more than two groups (e.g., culture type and admission status), overall differences were first evaluated using the Pearson chi-square test (or exact/Monte Carlo methods when appropriate). If the overall comparison was statistically significant, post-hoc pairwise comparisons were conducted using two-proportion z tests with Bonferroni correction; when sparse cells were present in pairwise tables, Fisher’s exact test was used. The corrected significance threshold for pairwise comparisons was set at *p* < 0.017. Statistical significance was defined as *p* < 0.05, and significant p values in tables were highlighted in bold. Because analyses were performed at the culture-sample level and some individuals could contribute more than one culture across different ED admissions, p values should be interpreted descriptively as comparisons of culture outcomes rather than patient-level causal inferences. All analyses were performed using IBM SPSS Statistics version 23.0 (IBM Corp., Armonk, NY).

### Power analysis

Because the study included all eligible cultures within the predefined 12-month period, no a priori sample-size calculation was performed. For completeness, we additionally performed a post-hoc assessment for the primary between-group comparison of contamination/commensal-flora reporting rates (pediatric vs. adult ED). Based on the observed proportions (35.5% vs. 23.0%), a two-proportion z test indicates very high statistical power at a two-sided alpha level of 0.05; however, this post-hoc metric should be interpreted descriptively and does not replace effect size estimation.

### Ethics

The study protocol was approved by the institutional ethics committee (Approval No: 2026/104, Date: 23.03.2026). The requirement for informed consent was waived owing to the retrospective design and anonymization of patient data.

## Results

**Fig. 1:**
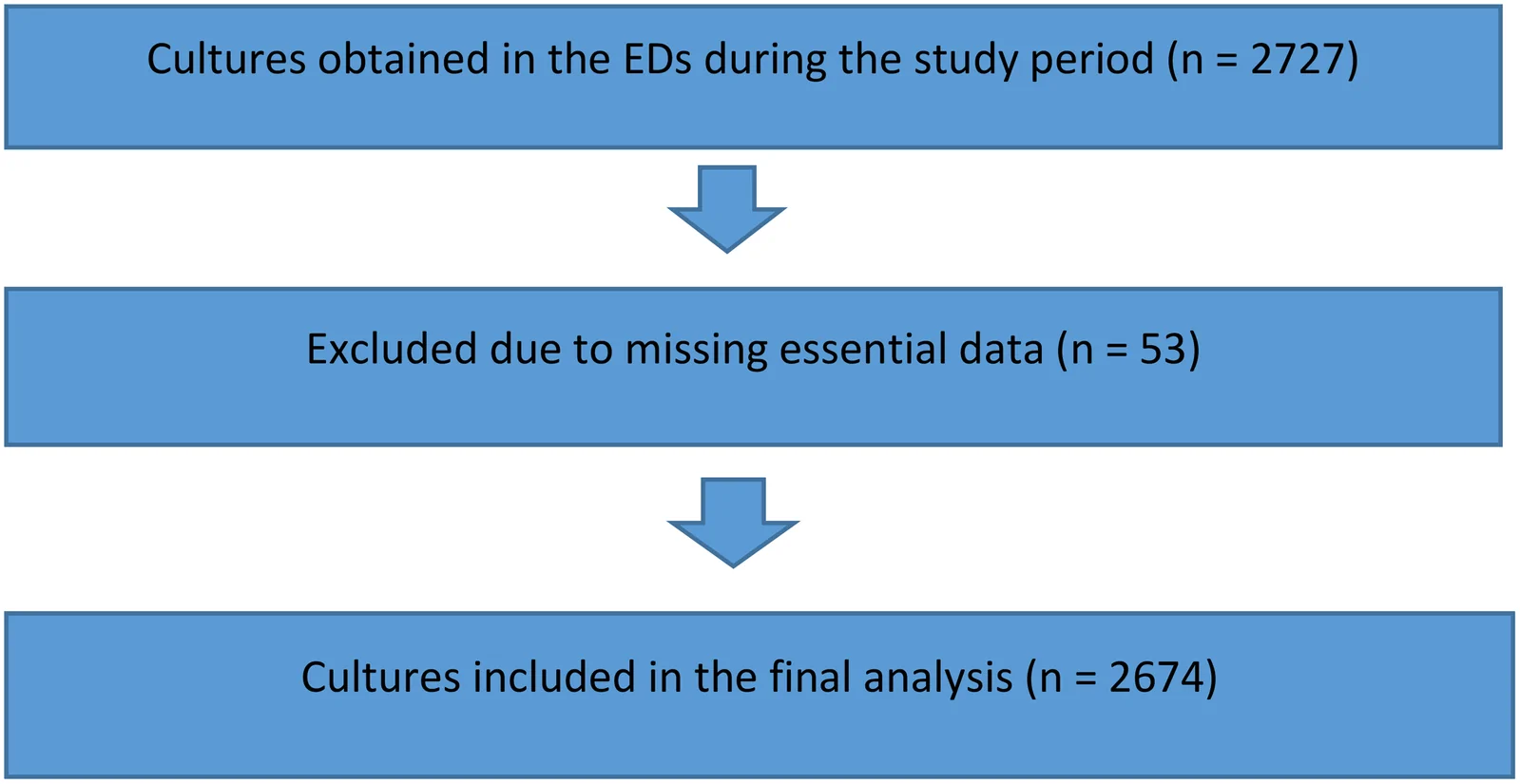
Flow diagram of the study population. All eligible cultures with complete data were included; no further exclusion criteria were applied

A total of 2727 cultures were identified during the study period. After excluding 53 records with missing essential data, 2674 cultures were included in the final analysis (Fig. 1). The median age of the patients was 66 years (25–75: 40–77), and 56.0% (1497) were male. Most cultures were obtained in the adult ED (90.1%), whereas 9.9% originated from the pediatric ED. More than half of the patients were discharged directly from the ED (52.8%), while 41.8% were admitted to the ward and 5.4% to the ICU. Urine cultures constituted the largest proportion of samples (56.7%), followed by blood cultures, whereas sterile body fluid cultures accounted for less than 1% of all specimens. These baseline characteristics are summarized in Table 1.

**Table 1:** General characteristics of cultures obtained in the emergency department

Variable	Value
**Age**,** years**	66 (40–77)
**Sex**	
Male	56.0 (1497)
Female	44.0 (1177)
**Department**	
Pediatric emergency	9.9 (265)
Adult emergency	90.1 (2409)
**Admission status**	
Discharged from ED	52.8 (1411)
Ward admission	41.8 (1118)
ICU admission	5.4 (145)
**Type of culture**	
Urine culture	56.7 (1516)
Blood culture (anaerobic)	23.3 (624)
Blood culture (aerobic)	19.2 (514)
Sterile body fluid	0.8 (20)

Continuous variables are presented as median (25–75), and categorical variables are presented as % (n)

After stratifying the cultures according to microbiological outcomes, nearly half of all samples showed no growth (48.8%). Contamination/commensal-flora, including laboratory reports consistent with normal skin or urogenital flora, was observed in 24.3% of cultures. Bacterial growth without extended-spectrum β-lactamase (ESBL) production was detected in 16.6% of samples, whereas ESBL-producing organisms were identified in 2.3%. Yeast growth was reported in 1.0% of cultures, and 7.0% of samples were rejected by the microbiology laboratory. Filamentous fungi, including Aspergillus spp. or Mucorales, were not recorded as a separate category in the available dataset. These results are presented in Table 2.

**Table 2:** Distribution of microbiological results of cultures obtained in the emergency department

Result category	Value
No growth	48.8 (1305)
Contamination/commensal-flora	24.3 (649)
Bacterial growth (ESBL-negative)	16.6 (443)
Sample rejection	7.0 (188)
Bacterial growth (ESBL-positive)	2.3 (62)
Yeast growth	1.0 (27)
Total	100 (2674)

Variables are presented as % (n)

The proportion of males was slightly lower among pediatric patients (49.1% vs. 56.7%, *p* = 0.020). Discharge from the ED was markedly more frequent in the pediatric group, whereas ward and intensive care admissions were more common among adults (all *p* < 0.001). Sample rejection and any microbiological growth were significantly higher in adult cultures, while contamination/commensal-flora was more frequent in pediatric samples. ESBL-producing bacteria were detected only in adult patients, whereas yeast growth was observed almost exclusively in adult cultures. These findings are summarized in Table 3.

**Table 3:** Demographics and culture outcomes in pediatric vs. adult emergency departments

Variable	Pediatric ED	Adult ED	*p*
**Age**,** years**	8 (4–12)	69 (53–78)	*<0.001
**Sex**			**0.020
Male	49.1 (130)	56.7 (1367)	
Female	50.9 (135)	43.3 (1042)	
**Admission status**			***<0.001
Discharged from ED	83.4 (221)	49.4 (1190)	
Ward admission	16.2 (43)	44.7 (1075)	
ICU admission	0.4 (1)	5.9 (144)	
**Sample rejection (yes)**	0.8 (2)	7.7 (186)	***<0.001
**Any growth (yes)**	6.4 (17)	21.4 (515)	**<0.001
**Yeast growth (yes)**	0.0 (0)	1.1 (27)	***0.159
**Contamination/commensal-flora (yes)**	35.5 (94)	23.0 (555)	**<0.001
**ESBL-producing bacteria (yes)**	0.0 (0)	2.5 (61)	***0.016

Continuous variables are presented as median (25–75), and categorical variables as % (n). The Mann–Whitney U test was used for continuous variables. Categorical variables were compared using the chi-square test; exact tests were applied when expected cell counts were < 5 (Fisher’s exact for 2 × 2 tables and exact/Monte Carlo methods for r×c tables). In the p column, * denotes p values from the Mann–Whitney U test, ** denotes p values from the chi-square test, and *** denotes p values obtained using exact tests. Statistical significance was set at *p* < 0.05, and significant p values are shown in bold

When cultures were evaluated according to sample type, urine cultures showed the highest proportion of any microbiological growth, followed by blood and body fluid cultures. Urine cultures also had higher rates of sample rejection and contamination/commensal-flora compared with blood and body fluid samples. Yeast growth was observed more frequently in urine cultures than in other sample types, whereas the overall distribution of ESBL-producing organisms did not differ significantly across culture categories. In post-hoc analyses with Bonferroni correction, most differences were driven by contrasts between urine and blood cultures. These results are presented in Table 4.

**Table 4:** Comparison of culture outcomes according to specimen type

Variable	Blood culture (*n* = 1138)	Urine culture (*n* = 1516)	Body fluid culture (*n* = 20)	*p*
Sample rejection (yes)	2.8 (32)ᵃ	10.3 (156)ᵇ	0.0 (0)ᵃᵇ	**< 0.001**
Any growth (yes)	17.0 (193)ᵃ	22.3 (338)ᵇ	5.0 (1)ᵃᵇ	**0.001**
Yeast growth (yes)	0.3 (3)ᵃ	1.6 (24)ᵇ	0.0 (0)ᵃᵇ	**0.003**
Contamination/commensal-flora (yes)	16.3 (185)ᵃ	30.4 (461)ᵇ	15.0 (3)ᵃᵇ	**< 0.001**
ESBL-producing bacteria (yes)	1.7 (19)	2.8 (42)	0.0 (0)	0.135

Variables are presented as % (n). Overall comparisons across culture types were performed using the Pearson chi-square test; exact (Monte Carlo) methods were applied when expected cell counts were < 5. When the overall p value was statistically significant, pairwise comparisons were conducted using Fisher’s exact test with Bonferroni correction (Blood vs. Urine, Blood vs. Body fluid, Urine vs. Body fluid), with a corrected significance threshold of *p* < 0.017. Superscript letters denote post-hoc differences: groups that do not share a letter differ significantly after Bonferroni correction

When the results were analyzed according to admission status, microbiological growth was more frequent among patients who required hospitalization compared with those discharged from the ED. The proportion of ESBL-producing bacteria also increased progressively from discharged patients to ward and ICU admissions. In contrast, contamination/commensal-flora was most common among discharged patients and less frequent among hospitalized patients. Sample rejection rates were lowest in discharged patients and highest in those admitted to the ICU. Post-hoc pairwise comparisons confirmed that most significant differences were observed between discharged patients and the two admission groups. These data are shown in Table 5.

**Table 5:** Comparison of microbiological findings according to admission status

Variable	Discharged (*n* = 1411)	Ward admission (*n* = 1118)	ICU admission (*n* = 145)	*p*
Sample rejection (yes)	4.2 (68)ᵃ	9.0 (101)ᵇ	12.4 (18)ᵇ	*<0.001
Any growth (yes)	14.6 (235)ᵃ	25.7 (287)ᵇ	30.3 (44)ᵇ	*<0.001
Yeast growth (yes)	0.6 (10)ᵃ	1.6 (18)ᵃᵇ	2.8 (4)ᵇ	**0.014
Contamination/commensal-flora (yes)	31.2 (502)ᵇ	20.8 (232)ᵃ	17.2 (25)ᵃ	*<0.001
ESBL-producing bacteria (yes)	1.4 (22)ᵃ	2.9 (32)ᵇ	5.5 (8)ᵇ	*0.003

Variables are presented as % (n). Overall comparisons across admission groups were performed using the Pearson chi-square test; exact (Monte Carlo) methods were applied when expected cell counts were < 5. When the overall p value was statistically significant, pairwise comparisons were conducted using two-proportion z tests with Bonferroni correction (Discharged vs. Ward, Discharged vs. ICU, Ward vs. ICU), with a corrected significance threshold of *p* < 0.017. Superscript letters denote post-hoc differences: groups that do not share a letter differ significantly after Bonferroni correction. In the p column, * indicates that the overall p value was obtained from the Pearson chi-square test, whereas ** indicates that it was obtained from exact (Monte Carlo) methods

## Discussion

In this study of microbiological cultures obtained in the ED, nearly half of all samples yielded no growth and approximately one quarter were classified as contamination/commensal-flora, including laboratory reports of normal/commensal flora. Such proportions indicate that a considerable share of cultures may have limited incremental clinical value at the population level, particularly when obtained outside well-defined indications. Previous ED studies have likewise reported that the diagnostic yield of blood cultures is generally low, with true-positive results representing only a small minority of samples [10]. A key point for interpretation is that our contamination/commensal-flora category was defined using laboratory wording (e.g., “mixed flora,” “commensal flora,” “normal skin/urogenital flora”) as a pragmatic indicator of low-interpretability or likely contaminated results, rather than organism-level adjudication. This category should not be interpreted as equivalent to commonly used blood culture contamination quality benchmarks. Formal blood culture contamination benchmarks generally require organism-level assessment, the number of positive bottles or culture sets, and clinical context, whereas our analysis was based on the wording of final laboratory reports. Accordingly, the 16.3% rate observed among blood cultures reflects the frequency of low-interpretability flora-related reports in routine ED practice rather than a strict blood culture contamination rate. For this reason, our estimates are not directly comparable to quality-benchmark contamination targets (e.g., < 2–3%), which rely on stricter contaminant definitions and typically require organism-level context [11]. Nevertheless, even within this laboratory-report–anchored framework, the high proportion of contamination/commensal-flora reports highlights a substantial operational burden of low-interpretability or ambiguous culture outcomes in the ED. Several analyses have shown that contamination is often higher in EDs than in other hospital units [12]. In some cohorts, contamination has constituted a substantial fraction of positive results [13], whereas standardized sampling protocols and targeted clinician training have been associated with meaningful reductions in contamination [14]. Taken together, these findings suggest that, although overall microbiological yield in the ED is modest, contamination-related reports represent a prominent and potentially reducible component of culture outcomes.

The relatively high proportion of non-diagnostic cultures in our cohort likely reflects factors intrinsic to ED practice. Emergency clinicians frequently make rapid decisions under diagnostic uncertainty, which may lower the threshold for obtaining cultures in patients with non-specific symptoms. Moreover, the practical challenges of achieving strictly aseptic sampling in crowded, time-pressured environments can further increase the risk of contamination. These observations support targeted quality-improvement strategies—such as clearer culture-ordering criteria, reinforcement of aseptic-technique training, and routine audit-and-feedback processes—to reduce unnecessary cultures, enhance microbiological yield, and ultimately improve patient safety.

Compared with adults, pediatric patients in our cohort exhibited a higher proportion of contaminated cultures and a lower rate of clinically meaningful growth. This pattern has been described in several previous reports and likely reflects a combination of biological, procedural, and organizational factors. In young children, specimen collection is often technically challenging because of smaller vessel size, greater movement, and limited cooperation, all of which may compromise aseptic technique and increase the risk of contamination [15]. Moreover, several studies have suggested that contamination rates may be higher in pediatric populations than in adults, even within the same institution, underscoring the vulnerability of pediatric sampling processes [13]. Collection methods commonly used in pediatric practice, particularly those that do not ensure a clean-catch urine specimen, may further contribute to false-positive results and unnecessary treatment [16]. Importantly, contaminated cultures in children are not benign; they have been associated with additional laboratory testing, prolonged antibiotic exposure, and increased healthcare costs [17]. Taken together, these observations highlight the importance of pediatric-specific quality-improvement efforts, including standardization of sampling techniques, use of age-appropriate collection devices, and clearer indications for culture acquisition in the emergency setting.

In our cohort, patients who required hospitalization had markedly higher rates of clinically meaningful culture growth, and ESBL-producing organisms became progressively more frequent from ward admissions to intensive care unit patients. This gradient likely reflects both greater illness severity and cumulative healthcare exposure. Hospitalized and critically ill patients are more likely to have advanced or complicated infections, prior or ongoing antibiotic therapy, invasive devices, and prolonged hospital stays, all of which can disrupt normal flora and create selective pressure favoring multidrug-resistant organisms, particularly in ICU settings [18, 19]. In addition, gastrointestinal dysfunction in critically ill patients may involve intestinal barrier disruption, dysbiosis, systemic inflammatory responses, and gut–organ axis interactions, which may facilitate microbial translocation and increase susceptibility to systemic infection in ICU settings [20]. From an ED perspective, these findings highlight the dual challenge of promptly identifying patients at genuinely high risk for infection while avoiding reflexive culture acquisition and indiscriminate broad-spectrum antibiotic use. Over-ordering cultures in low-risk patients can perpetuate a cycle of false-positive or contaminated results, unnecessary antibiotic exposure, and antimicrobial resistance, whereas thoughtful patient selection and integration of antimicrobial stewardship principles into ED workflows may help balance diagnostic certainty with responsible antibiotic use [21, 22].

When cultures were analyzed according to specimen type in our cohort, urine cultures demonstrated the highest proportion of microbiological growth compared with blood and sterile body fluid cultures. This pattern has been reported in other studies showing that urine cultures often have higher positive rates in ED populations, but also substantial contamination rates, reflecting both true urinary tract infections and challenges in specimen collection [23]. Urine culture contamination has been documented widely, and studies using interventions to improve clean-catch techniques have demonstrated that avoiding contamination remains a major quality challenge in both adult and pediatric settings [24, 25]. Midstream clean-catch urine cultures, the method most frequently used in ED practice, have shown contamination rates in the range of approximately 30–45% in real-world settings, emphasizing the need for better collection practices and patient education [24, 25]. In contrast, blood and sterile body fluid cultures tend to be ordered more selectively — usually in patients with stronger clinical suspicion of systemic or deep-seated infection — and are often obtained under more controlled aseptic conditions, which may partly account for their relatively lower contamination rates and different microbiological profiles [10]. In critically ill ED patients, prompt acquisition of blood or sterile body fluid cultures may remain essential for identifying pathogens in severe deep-seated infections, such as gas-forming pyogenic liver abscess, and for guiding targeted antimicrobial therapy [26]. Taken together, these findings underscore that specimen type influences both culture yield and contamination risk in the ED setting and that quality interventions tailored to specific culture types — especially urine specimens — may help optimize diagnostic performance, reduce unnecessary laboratory processing, and limit inappropriate antibiotic exposure.

The overall sample rejection rate of 7.0%, particularly the higher rejection rate observed among urine cultures, also deserves attention. Sample rejection in microbiology is generally related to preanalytical factors such as inadequate specimen volume, inappropriate containers, leakage, labeling errors, delayed transport, or noncompliance with collection requirements. Although the specific reasons for rejection were not consistently available in our retrospective dataset, the observed pattern suggests that preanalytical processes may represent an important target for ED-based quality improvement. Standardized specimen collection instructions, staff education, timely transport to the laboratory, and periodic feedback on rejected samples may help reduce avoidable sample rejection and improve diagnostic efficiency.

Although yeast growth was identified in a small proportion of cultures, filamentous fungi such as Aspergillus spp. or Mucorales were not recorded as a separate category in the available dataset. These findings should be interpreted cautiously. In the post-pandemic era, Aspergillus has gained renewed clinical importance, particularly among critically ill patients with severe viral pneumonia, COVID-19-associated pulmonary aspergillosis, prolonged ICU exposure, corticosteroid use, or other immunosuppressive conditions [27]. However, the present study was based on routine ED culture records and did not include targeted respiratory fungal cultures, fungal biomarkers, imaging findings, or clinical adjudication for invasive fungal disease. Therefore, the absence of a separate filamentous fungal category in our dataset should not be interpreted as evidence of absence of Aspergillus-related disease in the ED population.

Accordingly, our findings support a more selective, indication-driven approach to culture ordering in the ED, with prioritization of patients in whom results are most likely to change management (e.g., those requiring admission or with suspected bacteremia). In parallel, standardized collection protocols and periodic staff training may help reduce contamination and improve interpretability of culture results. Implementing ED-specific quality metrics (e.g., contamination/commensal-flora reporting rates by specimen type) could provide actionable feedback and guide targeted improvement efforts.

The incremental value of the present study lies in its broad, real-world assessment of ED culture practices across multiple specimen types rather than focusing solely on blood culture contamination or a single microbiological outcome. By simultaneously evaluating urine, blood, and sterile body fluid cultures in both adult and pediatric EDs, and by linking culture outcomes with patient disposition, this study provides an operational overview of where low-interpretability results, sample rejection, and microbiological positivity are concentrated in routine ED workflows. This descriptive perspective may help identify targets for diagnostic stewardship and quality-improvement interventions, even though patient-level clinical outcomes were not available.

This study has several notable strengths. First, it included all eligible cultures obtained over a one-year period in both adult and pediatric ED settings, which reduced selection bias and provided a comprehensive overview of routine microbiological testing patterns. Second, microbiology report terminology was standardized before analysis, helping reduce misclassification across heterogeneous laboratory result expressions. Finally, the study evaluated multiple operationally relevant culture outcomes, including contamination/commensal-flora reporting, ESBL production, yeast growth, and sample rejection.

This study has several limitations that should be considered when interpreting the findings. First, its retrospective design relied on routine electronic health record documentation, which precludes causal inference. Key clinical variables—such as presenting symptoms, comorbidities, validated illness-severity scores, and the timing of antimicrobial therapy relative to culture acquisition—were not consistently available and could not be assessed. This limits our ability to evaluate the clinical appropriateness of culture-ordering decisions and to determine whether early antimicrobial exposure contributed to the high “no growth” rates.

Second, our classification of “contamination/commensal-flora” was anchored to laboratory wording rather than independent investigator adjudication or organism-level context. While this approach reflects the operational burden of low-interpretability reports in the ED, the resulting rates may differ from strict quality-benchmarking definitions, particularly for blood cultures.

Third, our cohort included many older adults (median age, 66 years). For urine cultures, which constituted the largest share of samples, we could not reliably distinguish symptomatic urinary tract infection from asymptomatic bacteriuria, colonization, or contamination. This is particularly important in older populations, in whom positive urine cultures may not always indicate clinically significant infection. Because presenting symptoms, urinalysis findings, inflammatory markers, antimicrobial exposure, and medication history were not consistently available, clinical adjudication of positive urine culture results could not be performed.

Fourth, analyses were performed at the culture-sample level. Because some individuals contributed more than one culture across different ED admissions, observations were not fully independent; therefore, p-values should be interpreted descriptively as patterns in culture outcomes rather than patient-level inferences.

Fifth, urine collection method may also have contributed to the observed pediatric–adult differences. In our routine ED practice, urine samples are usually obtained by midstream clean-catch in cooperative adult patients, from urinary catheters in selected patients, and by urine collection bags in younger children when clean-catch sampling is not feasible. Because the exact collection method was not consistently available at the sample level, we could not quantify its effect on contamination/commensal-flora rates. Therefore, the higher contamination/commensal-flora rate observed in pediatric ED cultures should be interpreted cautiously, as it may partly reflect differences in urine collection methods rather than age group alone.

Sixth, specific reasons for sample rejection were not consistently available; therefore, we could not determine the relative contribution of inadequate specimen volume, inappropriate containers, leakage, labeling errors, delayed transport, or other preanalytical factors to the observed rejection rates.

Seventh, discharge status was obtained from ED disposition records and could not be further subclassified. Therefore, we could not distinguish routine discharge after clinical improvement from discharge against medical advice, referral to another facility, or other discharge pathways.

Finally, this single-center study was conducted at a tertiary care hospital, and local practice patterns and resistance epidemiology may limit generalizability to other settings. We also did not evaluate downstream clinical variables or outcomes, including antibiotic use, length of stay, readmission, mortality, or time to appropriate therapy, because these data were not consistently available in a structured format within the culture-sample-level dataset. Therefore, the present findings should be interpreted as descriptive patterns of ED culture outcomes rather than evidence of the clinical consequences of specific culture-ordering practices.

## Conclusion

In this retrospective analysis of emergency department cultures, nearly half of all samples showed no growth (48.8%), whereas almost one quarter (24.3%) represented laboratory-wording–based contamination/commensal-flora reports. These findings suggest that a substantial proportion of cultures may have limited diagnostic interpretability, particularly when obtained outside well-defined clinical indications. Culture performance varied by specimen type and clinical status: urine cultures exhibited both higher microbiological growth (22.3%) and higher contamination/commensal-flora rates (30.4%), while hospitalized—especially ICU—patients demonstrated greater microbiological positivity and a higher prevalence of ESBL-producing organisms. Taken together, these results highlight the need for more selective culture ordering, improved specimen-collection practices, and closer integration of diagnostic and antimicrobial stewardship principles into ED workflows. Prospective multicenter studies incorporating antibiotic exposure, length of stay, readmission, mortality, and time to appropriate therapy are warranted to clarify the clinical and economic consequences of ED culture practices.

## Data Availability

The datasets used and/or analyzed during the current study are available from the corresponding author on reasonable request.

## Abbreviations

ED: Emergency department
ICU: Intensive care unit
ESBL: Extended-spectrum beta-lactamase
CLSI: Clinical and laboratory standards institute
IDSA: Infectious diseases society of america
